# Abstracts and Selections

**Published:** 1908-08

**Authors:** 


### Abstracts and Selections.

Treasury Department,
Bureau of Public Health and Marine
Hospital Service.

Washington, D. C., July 9, 1908.

A board of commissioned medical officers will be convened to meet at the Bureau of Public Health and Marine Hospital Service, 3 B street SE., Washington, D. C., Monday, September 14, 1908, at 10 o'clock a. m., for the purpose of examining candidates for admission to the grade of assistant surgeon in the Public Health and Marine Hospital Service.

Candidates must be between 22 and 30 years of age, graduates of a reputable medical college, and must furnish testimonials from responsible persons as to their professional and moral character.

The following is the usual order of the examinations: 1, physical; 2, oral; 3, written; 4, clinical.

In addition to the physical examination, candidates are required to certify that they believe themselves free from any ailment which would disqualify them for service in any climate.

The examinations are chiefly in writing, and begin with a short autobiography of the candidate. The remainder of the written exercise consists in examination in the various branches of medicine, surgery and hygiene.

The oral examination includes subjects of preliminary education, history, literature and natural sciences.

The clinical examination is conducted at a hospital, and, when practicable, candidates are required to perform surgical operations on a cadaver.

Successful candidates will be numbered according to their attainments on examination, and will be commissioner in the same order as vacancies occur.

Upon appointment, the young officers are, as a rule, first assigned to duty at one of the large hospitals, as at Boston, New York, New Orleans, Chicago or San Francisco.

After four years' service, assistant surgeons are entitled to examination for promotion to the grade of passed assistant surgeon.

Promotion to the grade of surgeon is made according to seniority and after due examination as vacancies occur in that grade.

Assistant surgeons receive $1600, passed assistant surgeons $2000, and surgeons $2500 a year. Officers are entitled to furnished quarters for themselves and their families, or, at stations where quarters can not be provided, they receive commutation at the rate of thirty, forty and fifty dollars a month, according to grade.

All grades above that of assistant surgeon receive longevity pay, 10 per cent in addition to the regular salary for every five years' service up to 40 per cent after twenty years' service.

The tenure of office is permanent. Officers traveling under orders are allowed actual expenses.

For further information, or for invitation to appear before the board of examiners, address "Surgeon-General, Public Health and Marine Hospital Service, Washington, D. C."

